# Publication Landscape Analysis on Gliomas: How Much Has Been Done in the Past 25 Years?

**DOI:** 10.3389/fonc.2019.01463

**Published:** 2020-01-17

**Authors:** Chenzhe Feng, Yijun Wu, Lu Gao, Xiaopeng Guo, Zihao Wang, Bing Xing

**Affiliations:** ^1^Department of Neurosurgery, Peking Union Medical College Hospital, Chinese Academy of Medical Sciences, Peking Union Medical College, Beijing, China; ^2^Chinese Academy of Medical Sciences, Peking Union Medical College, Beijing, China

**Keywords:** gliomas, bibliometrics, machine learning, natural language processing, publication analysis

## Abstract

**Introduction:** The body of glioma-related literature has grown significantly over the past 25 years. Despite this growth in the amount of published research, gliomas remain one of the most intransigent cancers. The purpose of this study was to analyze the landscape of glioma-related research over the past 25 years using machine learning and text analysis.

**Methods:** In April 2019, we downloaded glioma-related publications indexed in PubMed between 1994 and 2018. We used Python to extract the title, publication date, MeSH terms, and abstract from the metadata of each publication for bibliometric assessment. Latent Dirichlet allocation (LDA) was applied to the abstracts to identify publications' research topics with greater specificity.

**Results:** We identified and analyzed a total of 52,625 publications in our study. We found that research on prognosis and the treatment of glioblastoma increased the most in terms of volume and rate of publications over the past 25 years. However, publications regarding clinical trials accounted for <5% of all publications considered in this study. The current research landscape covers clinical, pre-clinical, biological, and technical aspects of glioblastoma; at present, researchers appear to be less concerned with glioblastoma's psychological effects or patients' end-of-life care.

**Conclusion:** Publication of glioma-related research has expanded rapidly over the past 25 years. Common topics include the disease's molecular background, patients' survival, and treatment outcomes; more research needs to be done on the psychological aspects of glioblastoma and end-of-life care.

## Introduction

Gliomas are the most common tumor among adult primary malignant brain tumors (1). Traditionally, the World Health Organization (WHO)'s histopathological classification of glioma includes four grades: grades I and II are considered low-grade gliomas (LGG), and grades III and IV (glioblastoma-GBM) are considered high-grade gliomas (HGG) (2). In the past 25 years, researchers have made substantial progress in diagnosing and treating glioblastoma by explaining the molecular basis of primary brain tumors (3). This research on the molecular features of gliomas—including research on IDH, 1p/19q co-deletion, H3 Lys27Met, and RELA-fusion and other genetic parameters—has been incorporated into the 2016 WHO classification system (4). However, gliomas remain challenging to manage because of a lack of effective therapies.

Although a large amount of glioma-related research has been published, the trends and priorities of glioma research have not been adequately discussed among researchers. Bibliometrics are often used to perform quantitative analyses of academic literature. The number of academic publications on a given topic is often understood to reflect both researcher interest in and public attention to a topic at a given time (5). Previous bibliometric studies regarding gliomas have focused on specific gliomas and have only considered heavily cited articles (6, 7). Thus, the landscape of glioma-related research remains relatively underexplored and ill-defined.

Natural language processing (NLP) is a computational technique often utilized to analyze human language. NLP methods have yielded substantial research achievements; they can be and have been applied to process medical information (8, 9). For instance, researchers have applied NLP to identify specific themes of large publication data sets and thus investigate ongoing research trends. This has been done for research on cardiovascular systems and cancer rehabilitation (10, 11). Our study seeks to analyze the themes of glioma-related research that has been published in the past 25 years (1994–2018) and indexed in PubMed. Following previous bibliometric studies (5, 11), we used a machine learning methodology to conduct a more detailed analysis of glioma-related research topics. Our study examines the data for major trends in research instead of merely charting where and when most research has been published in order to help researchers understand the past and current status of glioma-related research, plan future research, and deliver more effective treatments to patients.

## Materials and Methods

We searched the public version of PubMed on April 1, 2019 for publications indexed with the MeSH term, “glioma,” limited by publication date from 1994 to 2018. The complete record of the search results was downloaded in XML format. We used Python to extract metadata from the original XML file, such as the year each article was published in, article abstracts, and articles' MeSH terms.

We used latent Dirichlet allocation (LDA) to identify the research topics in each article with greater specificity. LDA is a classic topic modeling method used in bibliometric studies to characterize large amounts of unstructured text. LDA creates a feature glossary of terms based on how often vocabulary words coexist in the document set. Once the glossary has been created, LDA determines the probability that an article examines a given research topic based on how often these glossary words appear in each document.

Our research used the abstracts of articles to model glioma-related research topics. We set the number of identified topics to 50. Based on the subject probability calculated by the algorithm, we identified the main topic of each article as defined by the topic with the highest probability. Through manual checks based on the abstract and MeSH terms, we named each term.

Finally, we used the Louvain algorithm for cluster analysis to establish a topic network, so as to better identify the relationship between these topics. For each article, we identified the two topics with the highest probability of attribution, counted how often each topic appeared in each document, and then established a link between the topics.

All the relevant Python code and the original publication dataset have been published on Zenodo (doi: 10.5281/zenodo.3382817) (Supplementary Materials 1, 2). Descriptive statistics were reported as mean ± standard deviation. The network visualizations in the article were performed with Excel and Gephi (https://gephi.org/) (12). Because this article is a bibliometric analysis, it does not require the approval of an institutional review board or ethics committee.

## Results

**Figure 1:**
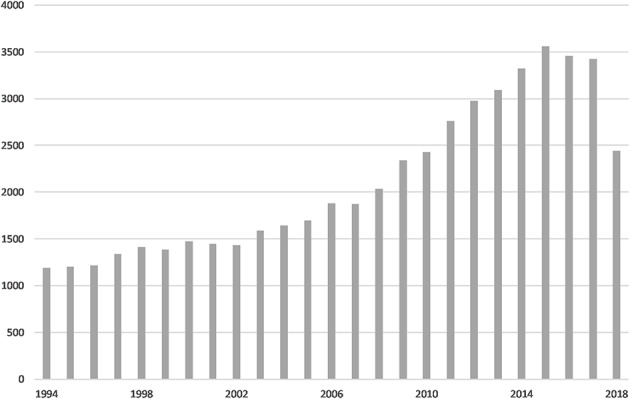
PubMed search results: articles per year.

The search string revealed 52,625 total publications. On average, 3,241 glioma-related research articles have been published per year over the past 5 years (Figure 1). There has been a decline in the number of articles published since 2015, especially in 2018; this may reflect an indexing delay, rather than a decline in research being conducted or in publication numbers. The results included 52,449 (99.67%) journal articles. These consisted of 1,619 (2.65%) clinical trials, 431 (0.82%) RCTs, and 846 (1.61%) multicenter studies. There were 6,035 (11.47%) case reports and 6,032 (11.46%) reviews.

### MeSH Analysis

After removing disease-specific words (such as *gliomas, glioblastoma*) and instrument-specific words (such as *Magnetic Resonance Imaging, immunohistochemistry*) from our MeSH terms, we divided the terms into two categories: one for MeSH terms referring to types of research subjects (e.g., *humans*), and one for all other remaining terms.

**Figure 2:**
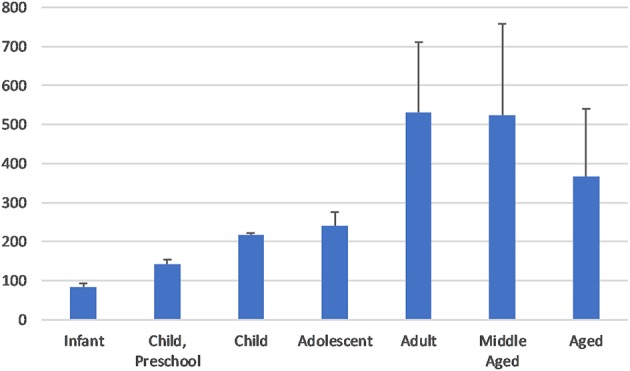
Annual output of glioma-related literature, broken down by age group.

Figure 2 illustrates the annual publication coverage by age of study populations. We identified seven age groups, defined as follows: infants (birth to 2–3 months), preschool children (2–5 years), children (6–12 years), adolescents (13–18 years), adults (19–44 years), middle-aged adults (45–64 years), and aged adults (65 years and above). Articles which involved subjects of more than one of these age groups were included in the total number of all corresponding age groups. In total, the adult and middle-aged adult groups featured most prominently in the publications reviewed by this study, followed by the aged adult group.

Table 1 shows the top 20 MeSH terms which appeared in retrieved articles aside from the subject-related MeSH terms mentioned above. The most popular terms include *neoplastic gene expression regulation, cell proliferation, prognosis*, and *apoptosis*. Table 2 shows the top 10 categories of glioblastoma research changes in each 5-years period. It is worth noting that the articles on *prognosis* and *antineoplastic agents* appear among the top 10 topics in every 5-years time period. Topics, such as *molecular sequence data* and *base sequence* ranked in the top 10 topics only during the first 5-years period (1994–1998). The expansion of glioma-related research publications mainly occurred under the MeSH terms *cell proliferation* and *gene expression regulation, neoplastic*. Over the past 10 years, research including MeSH terms, such as *signal transduction* and *tumor biomarkers* has attracted more attention.

**Table 1:** Overall ranking of research foci in the past 25 years.

**Rank**	**MeSH term**	**Record of occurrence in publications**	**% of total**
1	Gene expression regulation, neoplastic	5,358	10.18%
2	Cell proliferation	5,195	9.87%
3	Prognosis	5,099	9.69%
4	Apoptosis	4,807	9.13%
5	Antineoplastic agents	4,470	8.49%
6	Treatment outcome	4,385	8.33%
7	Signal transduction	4,010	7.62%
8	Biomarkers, tumor	3,167	6.02%
9	Combined modality therapy	3,096	5.88%
10	Neoplasm recurrence, local	3,056	5.81%
11	Cell movement	2,825	5.37%
12	Cell survival	2,722	5.17%
13	Temozolomide	2,604	4.95%
14	Dacarbazine	2,539	4.82%
15	Neoplasm invasiveness	2,529	4.81%
16	Survival analysis	2,460	4.67%
17	Survival rate	2,427	4.61%
18	Mutation	2,307	4.38%
19	Time factors	2,205	4.19%
20	Disease progression	2,034	3.87%

**Table 2:** Top 10 research foci in each 5-years period examined.

**Rank**	**First 5 years (1994–1998)**	**Second 5 years (1999–2003)**	**Third 5 years (2004–2008)**	**Fourth 5 years (2009–2013)**	**Fifth 5 years (2014–2018)**
1	Cell division 9.79%	Cell division 10.20%	Treatment outcome 9.34%	Cell proliferation 11.86%	Cell proliferation 17.77%
2	Combined modality therapy 7.79%	Apoptosis 8.20%	Apoptosis 8.89%	Gene expression regulation, neoplastic 10.84%	Gene expression regulation, neoplastic 15.03%
3	Molecular sequence data 7.13%	Prognosis 7.22%	Antineoplastic agents 8.85%	Prognosis 10.70%	Prognosis 12.26%
4	Neoplasm recurrence, local 5.87%	Antineoplastic agents 7.15%	Prognosis 8.30%	Treatment outcome 9.95%	Apoptosis 11.72%
5	Prognosis 5.79%	Treatment outcome 6.94%	Gene expression regulation, neoplastic 8.19%	Apoptosis 9.54%	Signal transduction 10.96%
6	Base sequence 5.79%	Combined modality therapy 6.38%	Cell proliferation 7.69%	Signal transduction 9.43%	Antineoplastic agents 9.60%
7	Antineoplastic agents 5.27%	Gene expression regulation, neoplastic 6.22%	Combined modality therapy 6.80%	Antineoplastic agents 9.17%	Treatment outcome 8.73%
8	Survival rate 5.24%	Survival analysis 5.69%	Neoplasm recurrence, local 5.81%	Biomarkers, tumor 6.88%	Biomarkers, tumor 8.40%
9	Time factors 4.30%	Time factors 5.66%	Survival analysis 5.79%	Dacarbazine 6.76%	Cell movement 8.29%
10	Survival analysis 4.25%	Neoplasm recurrence, local 5.59%	Signal transduction 5.77%	Temozolomide 6.75%	Neoplasm grading 7.78%

Table 3 shows the changes in the use of MeSH terms related to further treatment of gliomas in the literature over time. The results show that *combined modality therapy* and *antineoplastic combined chemotherapy protocols* have been the main themes of therapeutic research over the past 25 years. In addition, research on *neurosurgical procedures* has increased over the past 20 years. Moreover, the number of articles on the themes of *Molecular Targeted Therapy, Chemoradiotherapy*, and *Immunotherapy* have increased dramatically over the past 5 years.

**Table 3:** Top 10 research foci related to treatment in each 5-years period.

**Rank**	**First 5 years (1994–1998)**	**Second 5 years (1999–2003)**	**Third 5 years (2004–2008)**	**Fourth 5 years (2009–2013)**	**Fifth 5 years (2014–2018)**
1	Combined modality therapy 495	Combined modality therapy 468	Combined modality therapy 621	Combined modality therapy 880	Combined modality therapy 632
2	Antineoplastic combined chemotherapy protocols 201	Genetic therapy 326	Antineoplastic combined chemotherapy protocols 396	Neurosurgical procedures 549	Neurosurgical procedures 587
3	Genetic therapy 169	Antineoplastic combined chemotherapy protocols 254	Neurosurgical procedures 293	Antineoplastic combined chemotherapy protocols 487	Antineoplastic combined chemotherapy protocols 436
4	Radiotherapy dosage 156	Radiotherapy dosage 141	Genetic therapy 284	Genetic therapy 269	Chemoradiotherapy 374
5	Chemotherapy, adjuvant 112	Chemotherapy, adjuvant 124	Radiotherapy 200	Radiotherapy 227	Molecular targeted therapy 310
6	Radiotherapy, adjuvant 112	Radiotherapy, adjuvant 121	Radiotherapy dosage 177	Chemotherapy, adjuvant 223	Immunotherapy 247
7	Radiosurgery 73	Neurosurgical procedures 119	Chemotherapy, adjuvant 167	Radiotherapy dosage 203	Radiotherapy dosage 175
8	Radiotherapy 63	Radiotherapy 107	Radiotherapy, adjuvant 126	Radiotherapy, adjuvant 173	Radiotherapy 164
9	Immunotherapy 31	Radiosurgery 101	Radiosurgery 118	Chemoradiotherapy 167	Genetic therapy 148
10	Radiotherapy planning, computer-assisted 19	Immunotherapy 72	Immunotherapy 105	Immunotherapy 159	Chemotherapy, adjuvant 145

**Figure 3:**
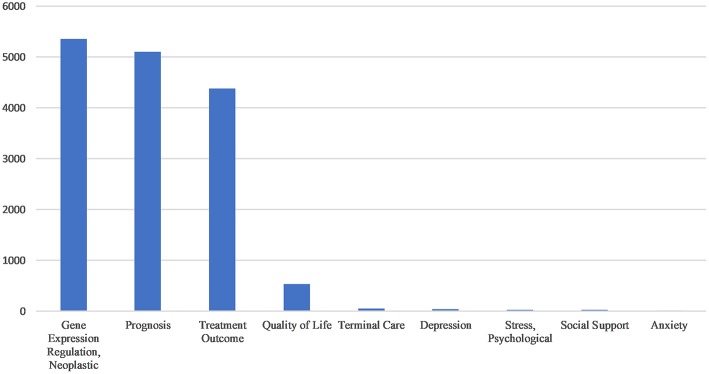
Comparison between the total amount of *neoplastic gene expression regulation, prognosis, treatment outcome*, and some MeSH terms related to terminal care and psychosocial research.

Figure 3 shows that there are fewer MeSH terms encompassing end-of-life care, quality of life, and psychosocial factors of gliomas compared to the large number of studies on prognosis, treatment effects, and tumor regulation mechanisms.

### Latent Dirichlet Allocation Analysis

**Figure 4:**
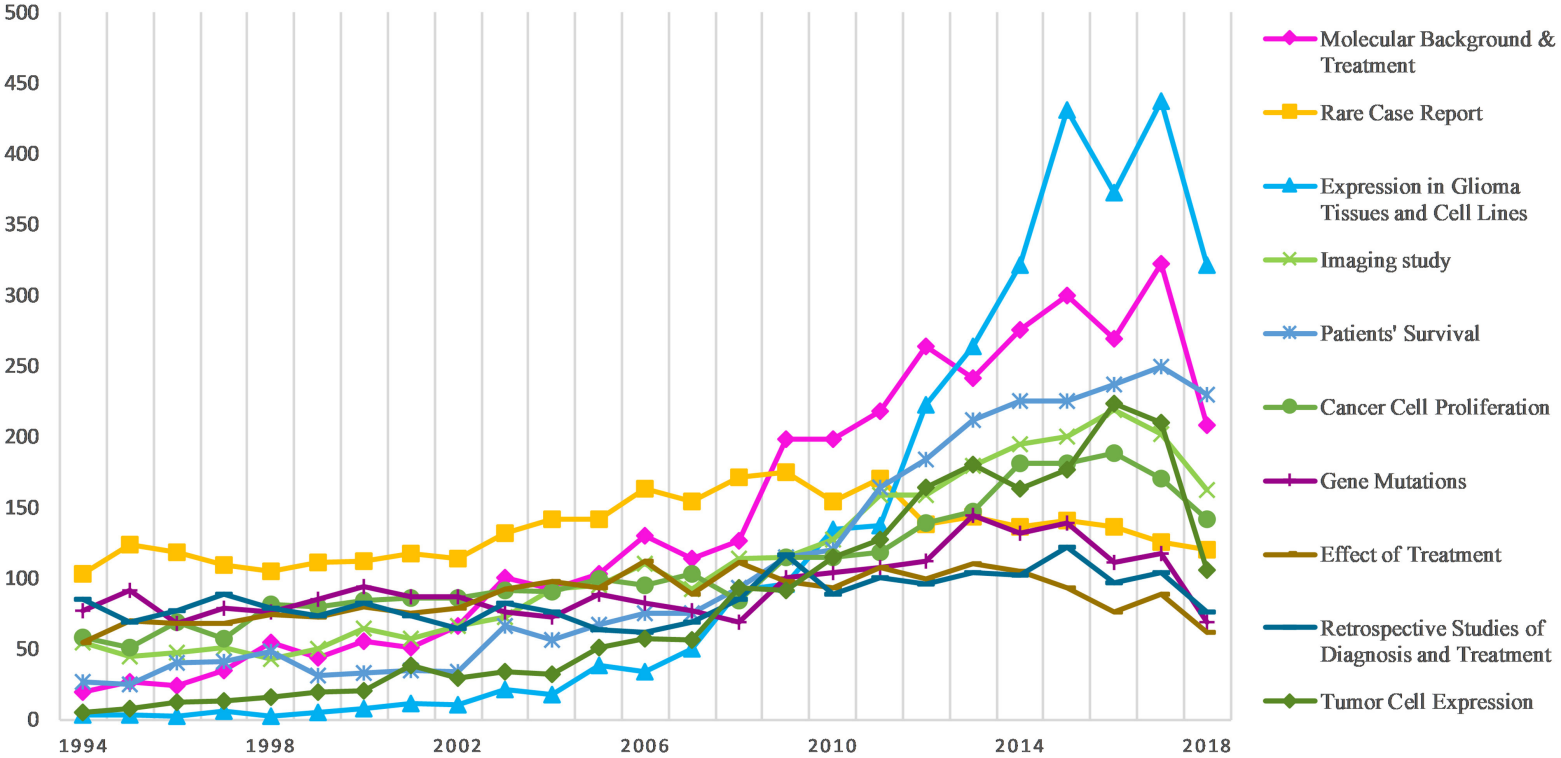
Latent Dirichlet allocation (LDA) analysis: increase in topic area articles per year.

LDA topics derived from the publications' abstracts provided further details on the research topics which have been covered most frequently in the literature. The top 10 LDA topics with the greatest volume changes over the past 25 years are shown in Figure 4. Of these, *molecular background and treatment, rare case report*, and *expression in glioma tissues and cell lines* were the LDA topics with the greatest volumes and rates of publication.

**Figure 5:**
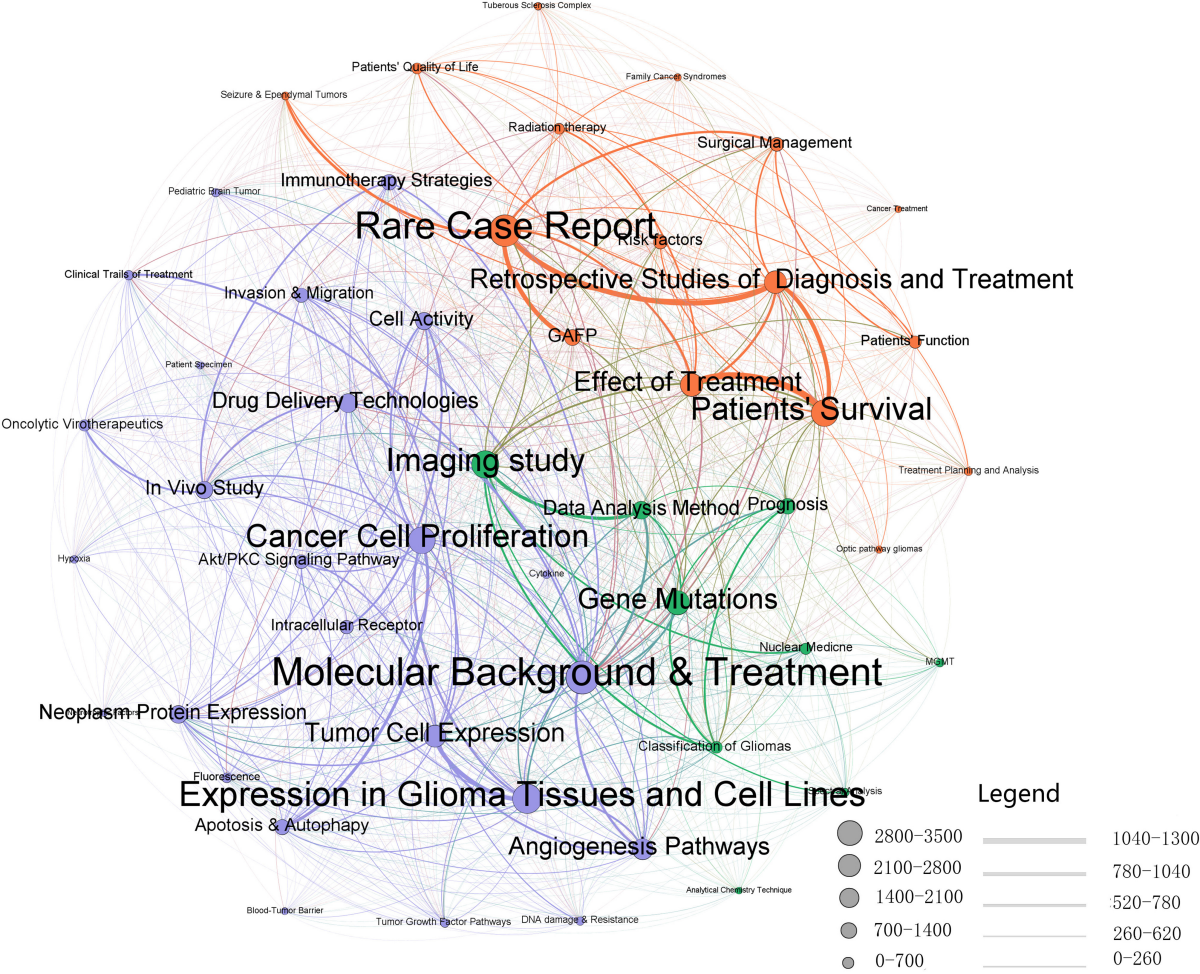
LDA research topic cluster network: inter- and intra-relationships. The purple cluster represents the “basic research” cluster, the green cluster represents the “imaging research” cluster, and the red cluster represents the “clinical research” cluster.

Research topic network analyses like ours highlight areas where clusters of inter-related topics co-occur, and thus provide insight into the relationships between prominent topics of interest (Figure 5). Three topic network clusters, each representing areas of strong intra-cluster relationships among publications, were identified by the Louvain method. They were: clinical research, basic research, and imaging research. For each topic, the size of the bubble represents the volume of characterization. The magnitude of the relationships among topics can be seen both within and between topic clusters by the network line connections. Thicker lines indicate that a high number of articles use common terms.

In the clinical research cluster, the most studied topics were *patients*' *survival, rare case report, effects of treatment*, and *retrospective studies of diagnosis and treatment*. Of these, *effects of treatment* and *patients*' *survival* show a strong association suggesting a strong emphasis on the efficacy of different treatment. The basic research cluster focuses heavily on *molecular background and treatment, expression in glioma tissues and cell lines*, and *cancer cell proliferation*. Strong relationships are seen across the three clusters regarding *molecular background and treatment*. The topics *imaging study* and *gene mutations* feature prominently in the imaging research cluster.

## Discussion

In 1994, spurred by the complexity of brain tumors and urgent need for treatment, the United States' National Cancer Institute (NCI) established a committee of the country's top neuro-oncologists to collaborate on novel treatment of brain tumors (13). Twenty-five years later, the literature on gliomas has grown substantially—it is now 44 times larger than it was in 1994. Significant, sustained growth is noted began in early 2000, and the literature has grown rapidly since 2010. The results of further LDA subject research responses may be shaped by the aforementioned relationship between *molecular background and treatment* and the increased number of e*xpressions in glioma cells and tissues*. This increase may reflect some of the important nodes in glioma treatment and may be related to breakthroughs in basic research in the field.

Pharmaceutical innovation and approval can drive glioma-related research. Temozolomide—an important drug in the treatment of gliomas—was shown to improve survival in glioma patients in a 1999 study (14). Further studies confirmed that temozolomide can benefit patients with MGMT mutations in 2005 (15). In 2007, the drug bevacizumab was approved by the FDA for the treatment of GBM (16). Accelerated progress in other areas—for instance, in medical technology—may also accelerate the generation of literature on glioma. In 2005, NCI and the National Genome Research Institute began The Cancer Genome Atlas Project; in 2008, they published the identification of several key mutations involved in GBM (17). In 2006, the relationship between chromosome 1p/19q heterozygous deletion and oligodendroglioma was found (18, 19). Molecular classification of high-grade astrocytoma tumors is initially formed in this year (20). In 2009, IDH1/IDH2 was found to be a good prognostic factor for low-grade gliomas (21). These important findings may drive more and more detailed research in these and other areas.

Surgery is an important part of glioma treatment. We have found that in the past 10 years, the literature indexed in the MeSH term *neurosurgical procedures* has gradually increased. This may be due to a variety of factors. The breakthrough in research on the molecular pathology of glioma, under the guidance of the new guidelines, requires us to obtain a clear pathology through neurosurgical means to help with treatment choice, the planning of treatment and patient prognosis (4). Maximally resecting a tumor while retaining nerve function is also one of the most difficult problems in neurosurgery. Breakthroughs in intraoperative surgical techniques may have led to an increase in research; for instance, it has been proven that intraoperative nuclear magnetic technique can increase the total number of tumors without increasing the neurological deficits of patients (22). Intraoperative fluorescence imaging technology can significantly improve the total rate of tumor (23). In addition, reductions in post-operative neurological dysfunction have been achieved in awake opening surgery combined with computer-guided frameless stereotactic numbering and intraoperative cortical stimulation and repeated neurological and linguistic function assessment in the case of functional tumors (24).

Although the overall trend of glioma publications is positive, our more detailed thematic analysis shows that in this field there is a relative lag in the study of end-of-life care and psychosocial aspects of gliomas. This result is consistent with some previous studies (25), including previous bibliometric studies of heavily cited papers in glioblastoma research (6, 7). Although palliative and supportive care is mentioned in the glioma guidelines, the lack of research means that the details of this kind of care are often not described in the literature. At the same time, previous studies have shown that in high-grade glioma patients, there is a lack of end-stage care. Dr. Forest's research on Medicare-related databases shows that about 37% of patients do not have access to hospice care, and that of the patients who did receive end-of-life care, 89% were enrolled for more than 3 days and 77% were enrolled for more than 7 days (26). These 25 years of research lag makes long-term survival a big challenge for patients with high-grade gliomas. The nursing needs of patients with glioma are diverse, and in addition to these needs caregivers of these patients do not fully understand how to administer different aspects of quality of life and end-of-life care throughout the course of treatment. In the future, more research should pay attention to the nursing needs of glioma patients across different regions and cultures so that these aspects of care can be improved.

Our study shows that existing research has several biases. One of these biases is reflected by the type of articles published. Our study identified a large number of rare case reports, which contains nearly 11% of all the articles. In a similar study of insomnia, the proportion of medical records was reported to be 20%, but in our study, this proportion is relatively small (5). However, in contrast to many studies that change clinical practices, the results and impacts of case reports are difficult to quantify. According to Carsten's study, most medical reports on glioblastoma are not cited often, and only a few are heavily cited (27). But considering the educational significance of medical records, this part of the research is still very important.

It is also noteworthy that, in our research, the results of the MeSH keyword and the LDA theme model suggest that there are many topics on treatment and prognosis. However, research from clinical trials only accounts for a small proportion of the overall literature. Furthermore, research of clinical trials of all glioblastomas registered between 2005 and 2016 showed that, among the 417 confirmed clinical trials, 93% of clinical trials were in phases I or II, and 26% of all clinical trial patients participated in the phase III study. Of the eight completed phase III trials, only one reported positive results (28). Among patients with high-grade gliomas, there are long development times, insufficiently disseminated information, poor decision-making regarding traveling, and low patient participation (28). Blood-brain barrier penetration, redundancy of intracellular signaling pathways, tumor molecular heterogeneity, and lack of validated biomarkers have been fully concerned and thought to be the main obstacles in glioma research (29, 30). However, clinical trials for glioma patients require more scholarly attention and the results of these trials need to be reported in detail, regardless of whether their results are positive or negative.

There are several limitations to this study. First, there are other databases besides PubMed available for bibliometric research, including Web of Science, Scopus, and Embase. But it is worth noting that PubMed contains the highest quality peer-reviewed research and excludes irrelevant, non-peer-reviewed publications (31). If future researchers could explore publications in other medical science databases, they would be able to provide a more comprehensive and detailed account of the field than we have provided here. In addition, some recently published papers may not have appeared in our study because they had not yet been indexed by MeSH words. These are common limitations to publication studies (10, 11). Finally, the LDA themes and their connections in this study have been created by artificial intelligence, which presents machine-driven understanding. A deeper, detailed exploration of these topics could deliver more easily interpretable and more accurate results, and so caregivers can deliver more effective treatment.

## Conclusions

The publication of glioma-related research has expanded rapidly over the past 25 years. The most common topics include the disease's molecular background, patients' survival, and treatment outcomes, but research on the psychological aspects of the disease and end-of-life care lag behind and demand more attention.

## Data Availability Statement

All datasets generated for this study are included in the article/Supplementary Material.

## Author Contributions

CF, YW, and BX: conceptualization. CF: methodology. CF, YW, LG, XG, and BX: formal analysis. CF, ZW, and BX: investigation. CF: writing—original draft preparation. CF and BX: writing—review and editing. BX: supervision.

### Conflict of Interest

The authors declare that the research was conducted in the absence of any commercial or financial relationships that could be construed as a potential conflict of interest.

## Supplementary Material

Supplementary Material 1 Original data of gliomas publication.

Supplementary Material 2 Python code for analysis.
